# Impact of sample acquisition and linear amplification on gene expression profiling of lung adenocarcinoma: laser capture micro-dissection cell-sampling versus bulk tissue-sampling

**DOI:** 10.1186/1755-8794-2-13

**Published:** 2009-03-09

**Authors:** Eric W Klee, Sibel Erdogan, Lori Tillmans, Farhad Kosari, Zhifu Sun, Dennis A Wigle, Ping Yang, Marie C Aubry, George Vasmatzis

**Affiliations:** 1 Department of Health Sciences Research, Mayo Clinic, Rochester, MN, USA; 2 Department of Biochemistry and Molecular Biology, Mayo Clinic, Rochester, MN, USA; 3 Department of Laboratory Medicine and Pathology, Mayo Clinic, Rochester, MN, USA; 4 Department of Molecular Medicine, Mayo Clinic, Rochester, MN, USA; 5 Department of Surgery, Mayo Clinic, Rochester, MN, USA

## Abstract

**Background:**

The methods used for sample selection and processing can have a strong influence on the expression values obtained through microarray profiling. Laser capture microdissection (LCM) provides higher specificity in the selection of target cells compared to traditional bulk tissue selection methods, but at an increased processing cost. The benefit gained from the higher tissue specificity realized through LCM sampling is evaluated in this study through a comparison of microarray expression profiles obtained from same-samples using bulk and LCM processing.

**Methods:**

Expression data from ten lung adenocarcinoma samples and six adjacent normal samples were acquired using LCM and bulk sampling methods. Expression values were evaluated for correlation between sample processing methods, as well as for bias introduced by the additional linear amplification required for LCM sample profiling.

**Results:**

The direct comparison of expression values obtained from the bulk and LCM sampled datasets reveals a large number of probesets with significantly varied expression. Many of these variations were shown to be related to bias arising from the process of linear amplification, which is required for LCM sample preparation. A comparison of differentially expressed genes (cancer vs. normal) selected in the bulk and LCM datasets also showed substantial differences. There were more than twice as many down-regulated probesets identified in the LCM data than identified in the bulk data. Controlling for the previously identified amplification bias did not have a substantial impact on the differences identified in the differentially expressed probesets found in the bulk and LCM samples.

**Conclusion:**

LCM-coupled microarray expression profiling was shown to uniquely identify a large number of differentially expressed probesets not otherwise found using bulk tissue sampling. The information gain realized from the LCM sampling was limited to differential analysis, as the absolute expression values obtained for some probesets using this study's protocol were biased during the second round of amplification. Consequently, LCM may enable investigators to obtain additional information in microarray studies not easily found using bulk tissue samples, but it is of critical importance that potential amplification biases are controlled for.

## Background

Microarray gene expression profiling is extensively used to study the etiology of disease and identify differential expression between two states. This high-throughput technology simultaneously measures expression levels in thousands of transcripts, providing a snapshot of the molecular makeup of a sample. The resulting data are representative of the cumulative expression of all cell types found in the sample. To increase specificity of the microarray expression signatures, investigators have used laser capture microdissection (LCM) for cell-collection prior to microarray hybridization. LCM is a precise extraction method that targets and extracts single cells from a sample [1-7]. Using this technology, a homogeneous collection of thousands of cells can be acquired and used to generate an accurate gene expression profile for a target tissue.

The perceived benefit of using LCM cell-sampling instead of bulk tissue-sampling for microarray analysis is balanced by the additional time and expense this method requires. LCM enables the precise extraction of target cells from a tissue sample consisting of a heterogeneous mixture of cell types. Bulk tissue sampling is faster and less expensive than LCM, however, sampled tissues often consist of a mixture of target and contaminating cell types. The process of LCM cell selection often generates low yields of RNA. Consequently, LCM-coupled microarray studies will use an additional round of RNA amplification (linear amplification) prior to microarray hybridization [5,8-10], which in some instances has been shown to bias the resulting expression values [11,12]. Comparative studies can demonstrate biological and methodological differences in expression data obtained by LCM cell-sampling experiments and bulk tissue-sampling experiments. Such studies will be intrinsically dependent on the tissue-type evaluated, as the constituent mixture of cell-types comprising the tissue will affect the bulk tissue-sampling expression data. Results from these analyses can be used to guide future experimental design, increasing the return for the research investment.

This study describes the first known comparison of microarray data generated from lung adenocarcinoma tumor cells and adjacent normal cells, acquired by LCM cell-sampling and bulk tissue-sampling. Lung cancer is the leading cause of cancer death in the U.S. in both men and women [13]. Its heavy burden on society has fueled substantial research and led to the dissemination of multiple public microarray datasets generated by bulk tissue-sampling protocols. Laser capture microdissection enables the specific collection of malignant epithelial cells within the lung adenocarcinoma tumor samples, while minimizing contamination by benign cells, stromal cells, and cells associated with other lesions. The results presented in this study should provide investigators with the information necessary to critically assess the value of LCM-coupled microarray expression profiling of lung adenocarcinoma, and determine if this approach would benefit their research goals. These data also provide a context by which LCM-cell-sampled expression profiling can be evaluated and compared to existing bulk tissue-sampled studies.

Microarray expression data were generated from LCM-cell-sampled and bulk tissue-sampled specimens acquired from matched cases, for ten adenocarcinomas and six adjacent-benign samples. To provide a reference to assess potential bias arising from the additional amplification step, RNA from two adenocarcinomas and two benign samples, acquired by bulk tissue-sampling, were linear amplified and hybridized to microarrays. The direct comparison of expression values revealed a substantial number of probesets with significantly altered expression levels between the bulk and LCM datasets. Evaluation of the linear amplified bulk data, however, showed many of the alterations are likely caused by an amplification bias, and not by differences in the cell populations profiled. Conversely, a comparison of differentially expressed probesets (cancer vs. normal) identified in the bulk and LCM datasets, showed a considerably higher number of differentially expressed genes were found in the LCM data. Interpreted in context of the linear amplified bulk data, this observation appears to be predominantly unrelated to the amplification bias and suggests LCM increases the sensitivity of detecting differentially expressed genes in the microarray data.

## Methods

### Samples

Fresh frozen tissue samples were obtained from the Mayo lung tissue bank for ten patients with stage IIIA or IIIB adenocarcinoma of the lung and with surgical tumor resection. Normal lung tissue adjacent to tumor in six patients were also obtained from the Mayo lung tissue bank. All samples were fast frozen within 30 minutes after resection and stored in -80 °C. All cases were first reviewed by a pathologist (MC) for their diagnosis accuracy and adequacy for a microarray study. Normal and tumor samples were selected if there was sufficient material to microarray profile both laser capture microdissected samples and bulk tissue samples. The use of the tissue for the study was approved by Mayo Foundation Institutional Review Board.

### LCM

For each patient, a pathologist assessed an H&E stained slide from each frozen tumor block (and normal block if needed). Fifteen sections (10 μm thick) of the chosen tumor and normal blocks were cut in a cryostat and placed on slides by the Tissue and Cellular Molecular Analysis (TACMA) core facility (Mayo Clinic, Rochester, MN) under Rnase free conditions. Slides were kept at -80°C and immediately stained with cresyl violet using an Ambion LCM staining kit (#1935) protocol. The desired area of tissue was microdissected from the slide within 30 minutes of staining using Arcturus LCM caps and microscope. The cap was placed on a tube that contained 75 μl of digestion buffer (RLT with BME from the Qiagen RNeasy micro kit #74004). The tube was inverted so the buffer covers the cap, vortexed at a medium speed for one minute and incubated at room temperature for 5 minutes. The tube was then centrifuged and the cap discarded. The samples were stored at -80°C until ready for RNA isolation. This procedure was repeated on the next slide for the case until a total of 5000 pulses were captured for that case. Each pulse targeted 3–5 cells. Due to tumor cell pleomorphism, it is difficult to standardize the number of cells captured per pulse. The number of slides used per case varied from 2 to 10, as every case and every tumor block had different sized tumors.

RNA is isolated according to the Qiagen RNeasy micro kit protocol (Qiagen, Valencia CA) with the following modifications. All of the tubes from the same sample were pooled together and 75 μl of 70% ethanol was added for each tube in the pool. The sample was applied to the column 700 μl at a time and spun according to protocol until the whole sample was applied. The RPE step was repeated to get rid of excess salt that may have accumulated due to multiple applications of sample to the column (depending on how many caps were needed per sample). The column was incubated with elution water for 5 minutes prior to spinning. The amount of RNA in each case was quantitated using the Quant-iT™ RiboGreen kit (Invitrogen Carlsbad, CA). The quality of the RNA was assessed using an Agilent 2100 Bioanalyzer in the Advanced Genomics Technology Center (Mayo Foundation). RIN (RNA integrity number) of 7 or greater was required to proceed to the linear amplification step.

Ten nanograms of RNA was linear amplified using the Affymetrix protocol (Genechip Expression Analysis Technical Manual Section 2) (Affymetrix kit # 900432 and Ambion kit #1334). The cRNA yield ranged from 84 μg to 134 μg, with a mean of 120 μg, per sample. Of each sample, 15 μg was then hybridized to the Affymetrix HU 133 plus 2.0 chips in the Advanced Genomics Technology Center using the same protocol.

### Bulk

For the bulk samples, the same blocks had 50 μm of tissue cut by the TACMA lab directly into a tube for tumor cases and 1000 μm of tissue for normal. These samples were kept frozen at -80°C until RLT buffer was added. The samples were homogenized using a mechanical homogenizer for 30 seconds and the RNA isolated using the RNeasy kit protocol. Once the RNA was isolated, it was evaluated using the Agilent and Ribogreen assays described above. The 1.4 μg of sample was then processed and hybridized (15 μg) onto the HU 133 plus 2.0 chip. From four samples, an additional 10 ng of RNA was linear amplified (as described for LCM) and then processed on the microarray chips. The cRNA yields for these four samples ranged from 130 μg to 145 μg, with a mean of 136 μg.

### Data preprocessing and analysis

The Affymetrix U133 Plus 2.0 gene chips were read using a GeneChip Scanner 3000 7G. Raw expression CEL files were analyzed using the dChip invariant set normalization procedure, with PM-only modeling. Expression values were log2 transformed.

For all comparisons, *a priori *thresholds were imposed to restrict the analysis to large changes in expression occurring beyond the scope of background signal. Two selection criteria were used when comparing expression values from A and B:

(i) Δ_A, B _≥ log_2 _2  (denotes a 4× change in expression)

(ii) max(A, B) ≥ log_2 _8  (ensured the higher value exceeded background levels)

To estimate the effect acquisition methods have on the selection of differentially expressed probesets, concordance between bulk and LCM rank-ordered differential probeset lists was calculated. Concordance was used to measure the agreement in ordered lists based on an absolute threshold, where probesets ranked above the absolute threshold on both lists were considered "in agreement" and the remainder "not in agreement". Analysis was performed over a range of thresholds and the results plotted. Probesets were rank ordered by the magnitude of the difference in log2 expression values, while maintaining the same selection criteria used throughout the study (Δ ≥ log_2 _2; max exp value ≥ log_2 _8). Comparisons were initially conducted on the cases with linear amplified bulk samples, and subsequently performed on the complete dataset.

All statistical tests were computed using the R free software package .

### Probeset distance to 3' end of transcript

Using Affymetrix's NetAffx Annotation File (Aug, 2007), the corresponding RefSeq transcripts, where annotated, were identified for all probesets. The sixth probe from each probeset was sequence-compared to its RefSeq transcript. Probesets for which the sixth probe mapped to the corresponding RefSeq transcript with 100% identity, were selected for further analysis. The distance between the 5' end of the sixth probe sequence and the transcript 3' end, minus any terminal poly-A repeat, was computed in bases. Distance values were determined for two groups: (i) all probesets with an average expression greater than log_2_8, and (ii) all probesets with significant alterations in expression between bulk and linear amplified bulk samples. Density plots for the two groups were computed using the R 'density' function, and independence in the distributions tested using the Kolmogorov-Smirnov test.

For these probesets, the corresponding RefSeq transcript was scanned for poly-A repeats of at least six adenines in length at the 3' terminus.

## Results

### Comparison of expression data between sampling methods

**Figure 1:**
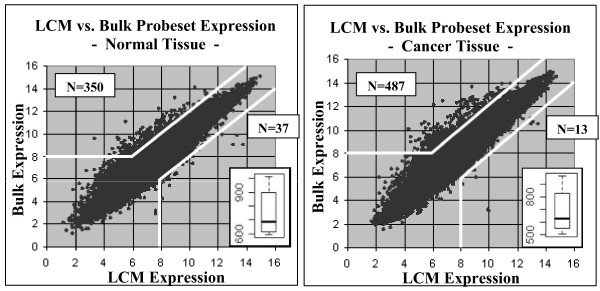
**The average probeset expression values in the LCM microarrays plotted against the average in the bulk microarrays, for normal and cancer tissues**. The selection thresholds for determining significantly varied expression are noted (white lines). Most variant probesets exhibited higher expression in the bulk microarrays versus the LCM microarrays, with 387 probesets in the normal tissue and 500 probesets in the cancer tissue displaying significantly different expression values in the LCM vs. bulk microarrays. The overlaid box-plots illustrate the distribution of variant probesets identified in the individual matched-case comparisons. No statistically significant differences in the number of altered probesets were found between normal lung tissue and lung adenocarcinoma.

The direct comparison of microarray data generated from bulk and LCM-sampled tumor and adjacent benign tissue identified a significant number of probesets with highly variant expression. All comparisons were limited to large changes in expression (4-fold or higher) to minimize type-I error arising from cross-hybridization and platform measurement variability. Results from the matched-sample comparisons between bulk and LCM data, stratified by cancer or normal status, are plotted in Figure 1. When comparing the bulk data to the LCM data, significant changes in expression levels were found for an average of 749 probesets across all six normal tissues samples and 684 probesets across all ten cancer tissues samples. Many consistent alterations were identified, with 114/749 probesets altered in all six normal tissue samples, and 153/684 probesets altered in all ten of the cancer tissue samples. The changes identified in normal and cancer tissues were compared using a Student's T-test (α = 0.05), with no statistically-significant difference identified (p = 0.46).

The preceding comparisons were repeated using two alternative methods to determine how robust the findings were. First, the average probeset expression value across all six bulk normal samples was compared to the average probeset expression value across all six LCM normal samples, and likewise for the cancer samples, identifying 387 probesets in normal tissue and 500 probesets in cancer tissue with significantly varied expression levels. Second, a Bonferroni corrected T-test (α = 0.05) was used to compare the microarray groups identified 577 probesets in normal tissue and 3616 probesets in cancer tissue, with significantly divergent expression.

**Figure 2:**
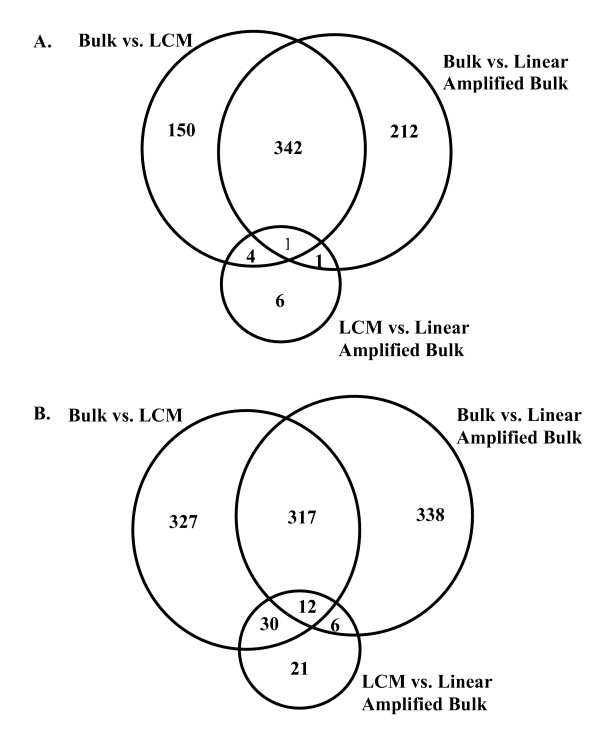
**Probesets with significant changes in expression level between bulk, LCM, and linear amplified bulk samples**. There is a similar distribution of overlapping probesets between the bulk samples and either of the amplified samples (LCM or Linear Amplified Bulk). Between amplified samples, there are very few probesets with significant changes in expression level, suggesting most of the changes observed between bulk and LCM are a by-product of an amplification protocol bias. These observations were consistent in both (A) normal tissue samples, and (B) cancer tissue samples.

To estimate whether the second round of RNA amplification, required for LCM sample processing, induced a bias in the expression data, RNA from two normal-bulk and two cancer-bulk tissue samples were linearly amplified. Using the two-sample average probeset expression values from the linear-amplified bulk, bulk, and LCM microarrays, the previous comparisons were repeated. As evident in Figure 2, very few probesets were identified with substantially different expression levels in the LCM samples compared to the linear amplified bulk samples. However, when comparing expression levels in the LCM samples to that in the bulk samples, or when comparing expression levels in the linear-amplified bulk samples with that in the bulk samples, there were substantially more divergent probesets identified. These observations were consistent in both the normal samples (Figure 2a) and cancer samples (Figure 2b). A further elaboration of these observations is presented in Additional file 1.

### Probeset distance to 3' end of transcript relative to gene polyA tail

**Figure 3:**
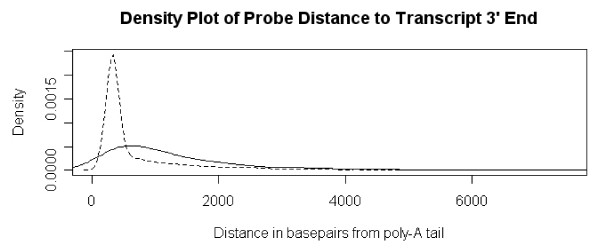
**Density plot of probeset distance to gene 3' end, for probesets with observed amplification bias (solid line) and a control set of all highly expressed (≥ log_2_8) probesets (dashed line)**. The biased probesets have a statistically different density distribution, shifted to the right, further from the gene's 3' end.

The second round of the linear amplification method used to process the LCM samples includes a random priming step which may induce a bias in the amplified expression level of probesets distant from the target gene's 3' end [14]. To test for this 3' bias effect, 318 probesets with expression levels significantly different in the bulk and linear amplified bulk microarrays (combined normal and cancer tissue), were evaluated. A total of 212/318 probesets possessed an Affymetrix annotated RefSeq transcript. The probeset to transcript 3' end distance was calculated and a density plot of the probeset distances (in bases) was generated (Figure 3). A second curve was generated for a control population of probesets with an average expression level greater than or equal to 8 (n = 12,634). Using a Kolmogorov-Smirnov test, the two density distributions were shown to be statistically different (p < 0.0001), with the distribution for the 212 selected probesets shifted to the right, further from the transcripts 3' end. False discovery analysis based on the selection of 100 random probeset populations of size 212, and compared to the control population, yielded Kolmogorov-Smirnov test parameters with a median p-value of 0.68 and a 90^th ^percentile p-value of 0.22. Based on the false discovery analysis, the increase in probeset distance from the gene 3' end observed for the probesets with linear amplification bias appears to be real and not an artifact of subset selection.

### Impact of LCM on identification of differentially expressed genes

A common objective of many microarray studies is the selection of differentially expressed genes; genes with significantly altered expression levels between normal and disease states. To determine whether LCM cell-sampling significantly effects the identification of differentially expressed genes, comparisons of up-regulated and down-regulated genes, identified using the bulk and LCM datasets, were carried out. Differential expression was computed using the average probeset expression across all cases. A total of 110 upregulated probesets were identified in the LCM dataset and 122 in the bulk dataset. Of these, 68 probesets were identified in both datasets. There were also 378 down-regulated probesets identified in the LCM dataset and 181 in the bulk dataset. Of these, 158 probesets were identified as down-regulated in both datasets. A list of the differentially expressed probesets identified in the bulk and LCM datasets is provided in Additional file 2.

**Figure 4:**
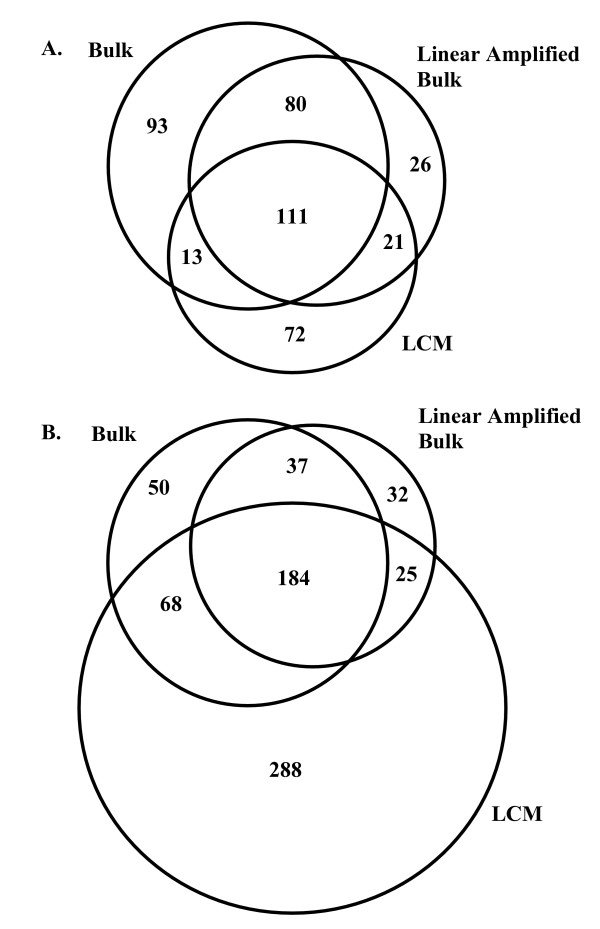
**Overlap of identified probesets with cancer to normal differential expression in the bulk, LCM, and linear amplified bulk datasets**. The number of upregulated probesets (a) identified is consistent between datasets, with the closest agreement between bulk and linear amplified bulk samples. For down-regulated probesets (b), there remains a tight association between bulk and linear amplified bulk samples. In the LCM samples the number of observed down-regulated probesets is substantially higher than that observed in either the bulk or linear amplified bulk samples.

To estimate the effect of the amplification process bias on the selection of differentially expressed probesets, the analysis was repeated using the two-sample average values for the bulk, LCM, and linear amplified samples. In the bulk data, 297 up-regulated probesets were identified, 238 in the linear amplified bulk data, and 217 in the LCM data. As illustrated in Figure 4a, 111 up-regulated probesets were found in all three datasets. There were substantially more probesets commonly identified between the bulk and linear amplified bulk samples, than between the bulk and LCM sample sets, or between the linear-amplified bulk and LCM sample sets. A total of 339 down-regulated probesets were identified in the bulk dataset, 278 in the linear amplified bulk dataset, and 565 in the LCM dataset. Of these, there were 184 probesets commonly identified as down-regulated in all three datasets (Figure 4b). A further elaboration of these observations is presented in Additional file 1.

To further evaluate the effect amplification bias has on the selection of differentially expressed genes, probesets expressed at significantly different levels in the bulk and linear amplified bulk samples were compared to probesets possessing cancer-to-normal differential expression levels in either the LCM or the bulk samples, but not both. The results are reported in Table 1. In general, the overlap between these two datasets was small. The largest overlap was for probesets uniquely identified as up-regulated in the bulk dataset. Detailed review of the individual expression profiles for these probesets revealed attenuated expression levels in the LCM and linear amplified bulk samples, suggesting the reason the probesets were not identified as upregulated in the LCM samples is a direct consequence of an amplification process bias (see Additional file 1 for more details).

**Table 1:** The overlap between the 603 probesets with clear amplification bias and probesets uniquely identified as upregulated or down-regulated in either the bulk or LCM dataset (but not both).

	Dataset Unique Cancer-to-Normal Differentially Expressed Probesets			
	Bulk Upregulated (N = 173)	Bulk Down-regulated (N = 87)	LCM Upregulated (N = 93)	LCM Down-regulated (N = 313)
Probesets with Identified Amplification Bias (N = 603)	38	6	1	10

### Impact of LCM on the ranking of differentially expressed genes

**Figure 5:**
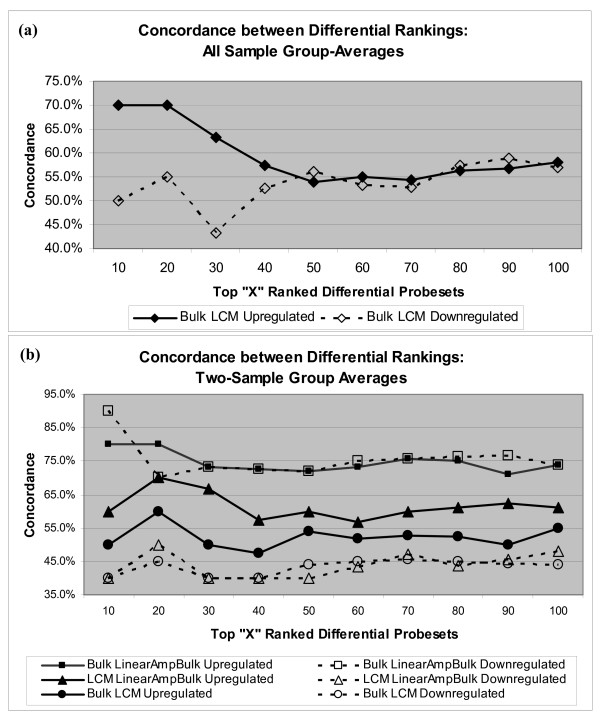
**The concordance of differentially expressed probeset rankings between (a) LCM cell-sampling and bulk tissue-sampling datasets at different levels of selection**. For the top 10 and top 20 ranked probesets there is high concordance in upregulated probesets (~70%). Concordance levels for both upregulated and down-regulated probesets converge to ~58% for the top 100 ranked probesets. For the LCM, bulk, and linear amplified bulk samples (b), there is consistently strong concordance between bulk and linear amplified bulk rankings of up- and down-regulated probesets (~75%). Concordance values are significantly lower for upregulated probesets between LCM and linear amplified bulk (~58%) or between LCM and bulk samples (~53%). The lowest concordance exists for the ranking of down-regulated probesets between LCM and either bulk or linear amplified bulk samples (~45%).

The impact LCM cell-sampling has on the relative rankings of differential probesets was also examined, by calculating the concordance between differentially expressed probeset rankings in the bulk and LCM datasets. A sliding threshold was used to select for the top 10, to the top 100 differentially expressed probesets. Probesets previously identified as up- or down-regulated were ranked by the magnitude of expression value change. The ranking concordance values were plotted against the selection thresholds, with independent response curves generated for up-regulated and down-regulated probesets. As illustrated in Figure 5a, the up-regulated probesets identified from the average expression levels across all cases, showed diminishing levels of concordance as the threshold for selection was increased. This trend was not observed for the down-regulated probesets. Concordance values for both upregulated and down-regulated probesets converged to approximately 55%.

The impact linear amplification has on the ranking of differentially expressed probesets was also evaluated. Response curves for rank concordance were computed for the differentially expressed probesets identified from the two-case average expression values in bulk, linear amplified bulk and LCM datasets (Figure 5b). The highest concordance (70%–80%) existed between bulk and linear amplified bulk datasets, for both up-regulated and down-regulated probesets. A smaller concordance was found for up-regulated probesets identified in the linear amplified bulk and LCM datasets (~60%). The concordance between up-regulated probesets identified in the bulk and LCM datasets was smaller yet (~50%). Concordance between down-regulated probesets identified in the LCM dataset and either the bulk or linear amplified bulk datasets, had the lowest values (40%–50%). In all measures of concordance, no consideration was given to the absolute rank; probesets were assigned a binary status of selected or not-selected according to their rank and the threshold being used. Overall, the concordance values reaffirmed the tight association between the bulk and linear amplified bulk differentially expressed probesets, and the lack of a linear amplification bias effect on the selection of differentially expressed probesets.

## Discussion

Comparative analysis of microarray data generated from LCM-cell-sampled and bulk tissue-sampled matched cases revealed many significant changes in expression levels between the two datasets. The discrepancies identified in these datasets highlight two main observations regarding the LCM process and resulting expression data. First, when the absolute gene expression levels are compared between LCM and bulk microarrays there are many significant differences identified. These variations in gene expression correlate with the second round of linear amplification, and do not appear to be related to the cell populations sampled. Second, substantially more down-regulated genes are uniquely identified in the LCM dataset than identified in the bulk dataset (Figure 4). This observation appears to be independent of the bias associated with the second round of amplification, and may reflect differences in cell population. An evaluation of replicate microarrays showed the inherent variability of the LCM amplified microarrays to be marginally higher than found for bulk tissue Affymetrix microarrays (see Additional file 1). While there have been several studies which evaluated LCM-coupled microarray analyses, none have reported the above observations in lung or lung adenocarcinoma [11,12,15-18].

The direct comparison of expression data from LCM-cell-sampled and bulk tissue-sampled microarrays identified a high number of genes (~650) with significantly different expression levels. To ascertain whether these changes are biologically significant, reflecting the expression patterns of the cell populations sampled, or an artifact arising from the LCM process, the expression of these genes in linear amplified bulk samples were examined. Approximately two-thirds of the variant probesets identified between the bulk and LCM datasets possess clearly attenuated expression levels in both the LCM and linear amplified bulk microarrays. The loss of expression signal in the linear amplified bulk data clearly indicates most of the observed variations in gene expression are a by-product of a bias arising from the amplification process. Only a small number of probesets displayed variant expression between the bulk and LCM microarrays, while displaying consistent expression between the bulk and linear amplified bulk microarrays. These probesets may measure biologically relevant variations of gene expression in the samples acquired by LCM cell-sampling and bulk tissue-sampling. However, these biologically relevant differences in expression are obscured by the process bias and not easily identified when directly comparing amplified (LCM) microarray data to unamplified (bulk) microarray data.

For most transcripts linear amplification is robust and reproducible. However, for a subset of probesets a bias is observed and may be at least partially explained by the nucleotide sequence priming chemistry used in the amplification procedure [14]. Transcripts are initially amplified using oligo dT primers from the 3' end. Following the first round of amplification, a second round of amplification is performed using random primers that hybridize anywhere along the length of the transcript, and initiate 5' to 3' sequence replication. This caused a disproportionate accumulation of sequences representing the 3' end of the transcripts compared with the 5' end. Longer transcripts yield more skewing of transcript levels in the 3' end and a higher 3'/5' ratios of *in vitro *synthesized RNA. Affymetrix constructed the U133 Plus 2.0 microarray with probesets located within 600 base pairs of the 3' end of the transcript . However, by mapping probeset positions to the target RefSeq transcripts, a subset of probesets are found substantially further than 600 base-pairs from the 3' terminus. A density distribution of probeset to transcript 3' terminus distance, for probesets with linear amplification bias and for a control group of significantly expressed probesets, illustrates a statistically significant shift in the distribution curves (Figure 3). It is reasonable to conclude from these observations that probeset to gene 3' end distance affects the validity of linear amplified expression values. It is also reasonable to conclude the biased amplified expression values do not occur at random, but with a high-likelihood in the subset of probesets located distant from the gene 3' end. Therefore, any study using a linear amplification protocol with random second round priming, should conduct a pilot study to identify and correct for potential process bias.

The identification of genes with differential expression between cancer and normal tissue is a common objective of microarray studies and therefore was examined in this study. The perceived value associated with LCM sampling of a more specific population of cells than obtained by bulk sampling, is realized in the selection of differentially expressed probesets. It is clear from the comparisons between the bulk, LCM, and linear amplified bulk datasets, LCM cell-sampling significantly effects the identification of differential expression. The effect is most pronounced for probesets with down-regulated expression in the cancer samples. Approximately twice as many down-regulated probesets are found in the LCM datasets, as found in either the bulk or linear amplified bulk datasets. The fact that this effect is not observed in the linear amplified bulk microarrays supports the conclusion that this is reflective of the cell population sampled and not a prominent by-product of the previously discussed amplification bias. This is clearly illustrated in Figure 4b, where the linear amplified findings align closely to the bulk findings, but are quite different than those obtained from the LCM data. The conclusion is further supported by the results presented in Table 1, regarding comparisons made to illustrate if the amplification bias was driving the unique identification of up- or down-regulated probesets in either the bulk or LCM datasets. Of the 313 down-regulated probesets found in the LCM samples and not in the bulk samples, only 10 exhibited strong amplification bias. The largest impact of the amplification bias was observed in the upregulated probesets identified only in the bulk dataset, where 38 of the 173 were affected. Examination of the individual expression profiles for these probesets reveals low expression values in the amplified samples. It is apparent the increased expression in the cancer tissue, observed in the bulk microarrays, is absent in the amplified samples as a result of the amplification process bias.

Individual expression profiles for those probesets uniquely identified as down-regulated in the LCM dataset were examined in the bulk and linear amplified bulk datasets. In most instances, the bulk and linear amplified bulk expression profiles showed some down-regulation, simply not as prominent as that observed in the LCM datasets. Subsequently, these probesets were not counted as down-regulated using the stringent criteria imposed in this study. These observations imply LCM cell-sampling provides a more sensitive method for identifying transcripts which lose expression in cancer. Conversely, it could be interpreted as the bulk tissue-sampling results in a partial masking of the lost signal in the bulk dataset by background expression in the contaminating cells. This background expression signal would attenuate the level of down-regulation observed in the bulk and linear amplified bulk datasets, leading to the skewed number of down-regulated genes identified in the bulk and LCM datasets.

To provide a practical assessment of the impact LCM cell-sampling has on the selection of candidate biomarkers, concordance of the relative rankings of differentially up- and down-regulated genes were evaluated. The impetus for this analysis lies in the limited capacity of most biomarker studies to validate and advance numerous discoveries. While some projects are using more sophisticated criteria for prioritizing biomarkers (i.e. systems biology, pathways, genetic and epigenetic associations), many investigators continue to use rankings based on the magnitude of expression variation to prioritize candidate gene lists. By determining the percent agreement between LCM and bulk datasets, at various thresholds, it becomes clear the differences in the differential genes selected are fairly consistent throughout the rankings. As Figure 5a illustrates, the concordance between LCM and bulk rankings is approximately 55%. The only significant deviation from this is in the highest ranked (top 20) up-regulated genes, where a stronger concordance exists (~70%). Concordance with the linear amplified bulk dataset, illustrated in Figure 5b, continues to show a consistent trend of tight agreement between the bulk and linear amplified bulk samples (~75%), with significantly lower correlation between the two amplified datasets (40%–60%). This supports the conclusion LCM cell-sampling provides a unique molecular vantage in the selection of differentially expressed genes, reflective of the cell population sampled.

The impact of LCM cell-sampling on the identification of differentially expressed probesets observed in this study has been reported in other cancer studies [12,16,17]. A study evaluating gene expression of in ER-α+ and ER-α-breast cancer tumors found 30% more differential genes using LCM cell-sampling than using bulk-tissue sampling [16]. This difference is not as strong as observed in this study, but it did involve the comparison of two sub-types of tumor and not cancer to normal tissue. The Chinnaiyan group found that LCM cell-sampling in prostate minimized the strong contaminating influence of stromal components and positively effected the selection of down-regulated genes [17]. These results very closely reflect what was observed in this study for lung, where the LCM cell-sampling appears to minimize the noise level caused by expression in contaminating cellular components. A third study, on rectal carcinoma, found bulk tissue-sampling to be more advantageous than LCM cell-sampling [12]. A strong amplification bias was reported on the gene expression profiles and a minimal effect of stromal contamination on the expression data. These findings may reflect tissue-specific effects, or reflect what comparisons were preformed. The authors used cluster analysis to determine many of the expression changes observed in the study, and never did comparisons on the selection of differential expression in the bulk and LCM datasets. By not performing these comparisons, there was no method for normalizing against amplification bias (by comparing amplified-to-amplified data, with non-amplified-to-non-amplified data) and identifying the underlying cell-sampling value found in the results presented in this manuscript.

An interesting observation can be made when comparing the results obtained by using different methods to directly compare the bulk and LCM microarray data. When defined thresholds are used to identify changes in expression between matched-sample microarrays, an average of 749 variant probesets were found in the normal samples and 684 in the cancer samples. Similar but an expectedly lower number of changes were found when comparing group-average expression values (382 normal, 500 cancer). However, when the same comparisons were repeated using a Bonferroni corrected T-test, a similar number of variant probesets was identified in the normal tissue (577), but considerably more were found in the cancer tissue (3616). It appears this may be partially explained by the relative sample size of the normal (n = 6) and cancer (n = 10) sample sets. When a random selection of six cancer sample-pairs were analyzed using the Bonferroni-corrected T-test, the number of variant probesets decreased and approached the number found in the normal tissue and by the defined threshold method (data not shown). The sensitivity of the Bonferroni-corrected method to sample size suggests a propensity towards identifying modest changes in expression. With the relatively noisy measurements obtained from microarrays, there is the risk these modest changes in expression may fall within the error bounds of the microarray platform, and be biologically irrelevant. The defined threshold method used in this study was specifically designed with conservative criteria, limiting selection to large, reproducible, changes in expression, which are more likely to be experimentally "real", and subsequently more robust to changes in sample size.

The linear amplified bulk microarray data generated in this study may have more universal application to researchers. Throughout these analyses, this data was used to estimate the impact of the T7 amplification bias on the expression values obtained from bulk tissue sampling and LCM cell sampling (with amplification). While this study specifically addressed lung samples, there may be broader applicability of the amplified bulk expression data. Any study using a similar amplification protocol coupled to LCM-based microarray analyses may find this data useful in interpreting the results. The amplification bias described in this manuscript should be tissue-type independent. Therefore, provided the gene under study is expressed in the nascent sample, this data may be used to test whether an apparently down-regulated transcript was reflecting a potential amplification bias or a modified expression state.

## Conclusion

This study comparatively analyzed expression data generated from lung adenocarcinoma and adjacent normal tissue acquired from the same samples using LCM cell-sampling and bulk tissue-sampling. The results highlight the importance that for any expression profiling study using LCM cell-sampling, steps are taken to evaluate all possible bias arising from the sample processing methods. It is also evident from these analyses that restricting any evaluation of acquisition methods to the comparison of absolute expression level changes between the methods is insufficient. The value of the acquisition method may only be observed when relative changes in expression values are compared. In this study, all comparisons were predicated on large changes in expression values. It is possible more value may be realized in LCM cell-sampling when the dynamic range of expression profiling technologies improve and low level changes can be confidently evaluated.

## Competing interests

The authors declare that they have no competing interests.

## Authors' contributions

EK was involved in the study conception, carried out the bioinformatics analysis, and drafted the manuscript. SE carried out the pathology review of all samples. LT carried out all the molecular biology and assisted in writing the manuscript. FK, ZS, DW, PY, MC were all instrumental in the study design, review of the ongoing study, and critical assessment and review of the manuscript. GV was involved in the study conception, study management, and critical assessment of the manuscript. All authors read and approved the manuscript.

## Pre-publication history

The pre-publication history for this paper can be accessed here:



## Supplementary Material

Additional file 1 **In depth discussion of specific examples of differentially expressed probesets.** It also contains additional discussions of the probe expression level bias identified in the manuscript. Click here for file

Additional file 2 **Contains a tab-delimited list of differentially expressed probesets identified in this study.** Click here for file
