# Supplementary material for: Impact of sample acquisition and linear amplification on gene expression profiling of lung adenocarcinoma: laser capture micro-dissection cell-sampling versus bulk tissue-sampling
Source: BMC Med Genomics. 2009 Mar 9;2:13. doi: 10.1186/1755-8794-2-13 (PMC2667433; doi:10.1186/1755-8794-2-13)
Supplement: Additional file 1 — In depth discussion of specific examples of differentially expressed probesets. It also contains additional discussions of the probe expression level bias identified in the manuscript. [file 1755-8794-2-13-S1.doc]

**Additional file 1**

**Comparison of differential expression profiles between datasets**

To determine whether LCM cell-sampling significantly effects the identification of differentially expressed genes, comparisons of up-regulated and down-regulated genes, identified within the bulk and LCM datasets, were carried out. What follows is a review of the individual expression profiles for probesets representative of the different expression profiles found within the bulk and LCM datasets, and their relationship to the profile found within the linear amplified bulk dataset.

*Down-regulated Genes*

**Supplemental Figure 1**

**Bulk**

**Amplified**

**Bulk**

**LCM**

1

1

1

2

2

2

3

3

3

4

4

4

*All bulk and LCM matched-cases are ordered from left to right. The linear amplified bulk sample cases are denoted with numeric superscripts, in all three datasets.*

MEOX2 displays significant down-regulation in the LCM cell-sampled dataset. In the bulk tissue- sampled dataset, MEOX2 also is down-regulated, but the change in expression is not has large as that observed in the LCM dataset. There is no evidence from the linear amplified bulk data, that the loss of expression is the product of an amplification bias, as its expression profile very clearly mimics that observed in the bulk dataset. This attenuated change in expression observed in the bulk dataset could be explained by low or absent expression of MEOX2 in the “contaminating” cell types, thereby diluting the overall per-cell average expression of MEOX2 in the bulk normal samples, and artificially reducing the differential expression between cancer and normal cells. MEOX2 is a growth arrest-specific homeobox, which is functionally supportive of the observation the transcript expression in lost in the cancer cells.

**Supplemental Figure 2**

**Bulk**

**Amplified**

**Bulk**

**LCM**

1

1

1

2

2

2

3

3

3

4

4

4

This probeset exhibits a strong down-regulated expression profile in the bulk and linear amplified bulk datasets, but a completely attenuated expression profile in the LCM dataset. It is clear from the illustration, that this attenuated expression is not the product of an amplification bias, as the amplified bulk samples reflect the same expression levels seen in the bulk corresponding bulk samples. It is therefore reasonable to conclude the LCM expression data is accurate and this probeset is expressed at low levels in the normal lung epithelial cells. Consequently, the higher expression values in the bulk dataset may reflect transcript expression in other “contaminating” cell types. A disproportionate representation of these other cells in the bulk cancer samples would create the false impression that this transcript is down-regulated in the tumor. This probeset maps to an intronic region between two ADAMTS9 exons. It is reasonable to conjecture 1556989_at measure the expression of an alternative form of ADAMTS9, expressed in non-epithelial components captured in the bulk tissue-capture process.

**Supplemental Figure 3**

**Bulk**

**Amplified**

**Bulk**

**LCM**

1

1

1

2

2

2

3

3

3

4

4

4

KAL1 is a uniformly down-regulated probesets with near identical expression profiles in all datasets. There is no evidence of linear amplification bias, nor any perceived value in the LCM data compared to the bulk data, for the identification of this gene.

*Up-regulated Genes*

**Supplemental Figure 4**

**Bulk**

**Amplified**

**Bulk**

**LCM**

1

1

1

2

2

2

3

3

3

4

4

4

The 1565817_at probeset targeting IKZF1 illustrates a case where upregulated expression is observed in the LCM dataset, but not in the bulk dataset. The bulk samples have a flat expression profile, at or below the background level. The linear amplified samples, however, have elevated expression in the normal samples, and even more elevated expression in the cancer samples, mimicking the up-regulation observed in the LCM dataset. For this probeset, it is difficult to surmise why the differential expression is only observed in the amplified samples. It may reflect a non-linear amplification of the transcript sequence, caused by some unusual sequence characteristics. It may also illustrate an effect that has been previously reported, where amplified samples appear to be more sensitive to the detection of differential expression, in low expression level transcripts [1].

**Supplemental Figure 5**

**Bulk**

**Amplified**

**Bulk**

**LCM**

1

1

1

2

2

2

3

3

3

4

4

4

ANLN expression profile illustrates a situation where transcript up-regulation observed in the bulk dataset is not observed in the LCM dataset. In this situation, the linear amplified bulk data has attenuated expression values in the cancer samples, and suggests the absence of upregulated expression in the LCM dataset is a by-product of the amplification process bias. Functionally, ANLN is involved in cytokinesis and a major component in maintaining the integrity and completion of the cleavage furrow. It is therefore not surprising that this cell-cycle gene is upregulated in the highly proliferative tumor cells. ANLN possess two probesets in the Affymetrix U133 Plus 2.0 microarray, 1552619_a_at which shows the attenuated expression in the amplified samples (illustrated above) and 222608_s_at which does not show any attenuated expression in the amplified samples. The 1552619_a_at probeset targets a region of the Refseq gene sequence 5’ of that targeted by 222608_s_at, supporting the idea that the further a probeset is located from the target gene’s 3’ end, the more likely it will be affected by the amplification bias.

**Supplemental Figure 6**

**Bulk**

**Amplified**

**Bulk**

**LCM**

1

1

1

2

2

2

3

3

3

4

4

4

TOP2A is a uniformly up-regulated probesets with near identical expression profiles in all datasets. There is no evidence of linear amplification bias, nor any perceived value in the LCM data compared to the bulk data, for the identification of this gene. The gene encodes for a protein involved in cellular proliferation and commonly seen over-expressed in tumors.

**Group comparison of probeset expression level between cell-sampling methods to access amplification bias**

To ascertain whether linear amplification introduces a significant bias to the probeset expression levels, causing the observed expression variations between sample sets, RNA from two normal-bulk and two cancer-bulk tissue samples were linearly amplified. Comparisons of probeset expression levels were then made between the two-sample average probeset expression levels in the bulk tissue samples, the LCM tissue samples, and the linear-amplified bulk tissue samples. Comparisons were made independently for normal tissue samples and cancer tissue samples, to remove the disease influence on the observations. As shown in Supplemental Table 1, comparing average probeset expression values in the bulk samples to either the LCM samples or the Linear Amplified Bulk samples results in a large number of probesets with significantly different expression levels (normal tissue: 479 bulk vs LCM; 556 bulk vs linear amp bulk). However, when comparing average probeset expression levels between the LCM and linear amplified bulk samples, the two sample types that underwent a second round of amplification, there are very few probesets with significantly different expression levels (normal tissue: 12 LCM vs linear amp bulk). This suggests that a majority of the observed changes in probeset expression levels between the bulk and LCM sample sets are an artifact of the second round of amplification and not a product of the cell sampling. These findings are consistent for both normal and cancer tissues.

**Supplemental Table 1: Number of probesets with significantly altered expression levels between sample types**

|  | **Sample Type** | **Bulk** | **LCM** | **Linear Amp Bulk** |
| --- | --- | --- | --- | --- |
| **Normal** | **Bulk** | -- | 497 | 556 |
|  | **LCM** | 497 | -- | 12 |
|  | **Linear Amp Bulk** | 556 | 12 | -- |
|  |  |  |  |  |
| **Cancer** | **Bulk** | -- | 686 | 674 |
|  | **LCM** | 686 | -- | 69 |
|  | **Linear Amp Bulk** | 674 | 69 | -- |

**Group comparison of cancer-to-normal differential expression between cell-sampling methods to access amplification bias**

A clear second round of amplification bias was identified in the expression level of probesets when comparing across sample types (bulk, LCM, linear amp bulk). Here, the amplification bias influence on the selection of differentially expression probesets, when comparing cancer to normal samples within a single sample-type, is evaluated. To estimate this effect, the number of common differentially expressed probesets identified in each sample type was tabulated. As shown in Supplemental Table 2, the amplification bias in the selection of differential probesets is small. In fact, opposite of what was observed previously, there appears to be a stronger congruency between the bulk and linear-amplified bulk samples, than between either and the LCM samples. A substantially larger number of up-regulated probesets were commonly identified between bulk and linear-amplified bulk samples, than between the bulk and LCM samples or between the linear amplified bulk and LCM samples. This trend is not observed in the overlap of common down-regulated probesets. However, there is a significantly larger number of down-regulated probesets identified in the LCM samples, compared to either the bulk or the linear amplified bulk samples. In both instances, the observations tend to suggest that the bulk and linear amplified bulk samples behave in a more congruent manner, than with the LCM samples.

**Supplemental Table 2: Number of probesets with significant differential expression (cancer vs normal) common between sample types**

|  | **Sample Type** | **Bulk** | **LCM** | **Linear Amp Bulk** |
| --- | --- | --- | --- | --- |
| **Up-regulated** | **Bulk** | 297 | 124 | 191 |
|  | **LCM** | 124 | 217 | 132 |
|  | **Linear Amp Bulk** | 191 | 132 | 238 |
|  |  |  |  |  |
| **Down-regulated** | **Bulk** | 339 | 252 | 221 |
|  | **LCM** | 252 | 565 | 209 |
|  | **Linear Amp Bulk** | 221 | 209 | 278 |

**Replicate analysis of microarrays**

Replicate analysis of the linear amplified microarrays was performed and compared to the correlation values computed from existing technical replicate analysis in bulk tissue specimens. Five samples were taken from a starting RNA extract, processed by linear amplification, and analyzed on microarray chips. We observed an average pair-wise correlation between the five replicate samples of 0.89 (SD 0.019). To compute pair-wise correlation in bulk tissue samples, raw cel-files were downloaded for five sample-A, technical replicates from the MAQC project [2]. These arrays were processed in the same manner as the LCM microarrays yielding an average pair-wise correlation of 0.92 (SD 0.003). It is important to note that the LCM samples included independent linear amplification steps, while the bulk samples were true technical replicates. This explains the slightly reduced pair-wise correlation for the LCM samples.

**Evaluation of 3’ distance on probeset intensity values and differential selection of tumor versus normal genes**

To provide another perspective on the effect 3’ distance has on probeset expression levels, independent scatter plots of log-intensities vs distances from probeset to 3' end were generated for the LCM, bulk and linear amplified bulk data (Supplemental Figure 7). There is a discernable tightening of the distribution of points in both the linear amplified bulk and LCM plots, compared to the bulk plot. This may reflect a small measure of the amplification bias reported in this study.

**Supplemental Figure 7**


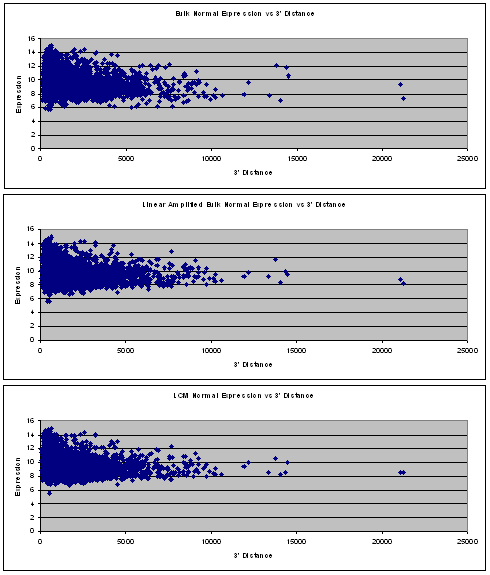


To further evaluate the role of the amplification bias versus cell population selection on the differences observed when selecting differentially expressed probesets in the LCM and bulk samples, box-plots of distance from probeset 3’end were created. Independent plots were created for up-regulated and down-regulated probesets identified in the LCM, linear amplified bulk, and bulk samples (Supplemental figure 8). The graph highlights a trend in the spread of the data points (regarding 3’ distance), that diminishes with both the amplification process and cell selection process. It also shows that the down-regulated probesets exhibit a tighter spread of data (regarding 3’ distance), and that for up-regulated probesets, there is a slightly larger variation versus 3’ distance than observed for down-regulated probesets.

**Supplemental Figure 8**


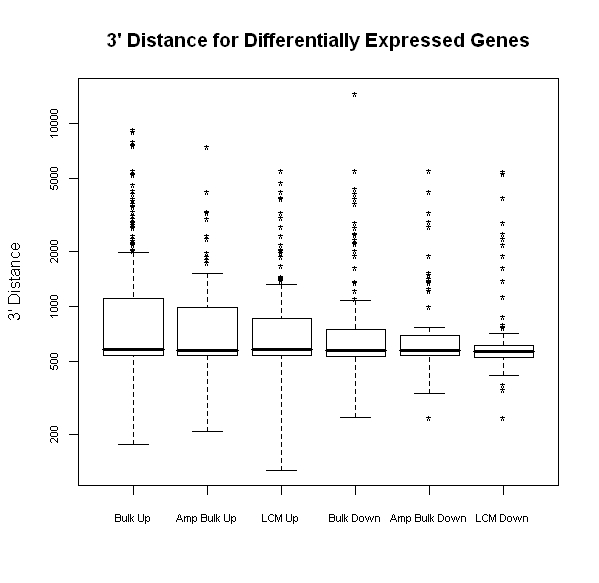


1. Polacek DC, Passerini AG, Shi C, Francesco NM, Manduchi E, Grant GR, Powell S, Bischof H, Winkler H, Stoeckert CJ Jr, Davies PF: **Fidelity and enhanced sensitivity of differential transcription profiles following linear amplification of nanogram amounts of endothelial mRNA*.***  *Physiol Genomics* 2003, **13**:147-156.
2. MAQC Consortium, *et al.*: **The MicroArray Quality Control (MAQC) project shows inter-and intraplatform reproducibility of gene expression measurements.** *Nat Biotechnol* 2006, **24**:1151-1161.
